# Ambiguity and free will: the topology of decision in quantum and quantum-like sciences

**DOI:** 10.1098/rsta.2024.0379

**Published:** 2025-11-27

**Authors:** Arkady Plotnitsky

**Affiliations:** ^1^ Literature, Theory and Cultural Studies Program, Philosophy and Literature Program, Purdue University, W. Lafayette, IN, USA

**Keywords:** ambiguity, complementarity, free will, reality without realism, topology

## Abstract

This article considers the relationships between quantum theory (QT) and quantum-like theories (QLTs), theories using mathematical models based on the formalism of QT, from a reverse perspective, that of QLTs. The article argues that QT is no longer a theory of the behaviour, in particular motion, of physical objects, as was the case in classical physics and relativity. Instead, QT is a form of decision theory, involving a special ‘topology’ of decisions, using the term topology in part metaphorically, but only in part, because it applies in its proper mathematical sense to the formalism of QT. Part of this topology is the concept of free will, reconsidered through the concept of decision. This character of QT is grounded in a particular type of interpretations of QT, ‘reality without realism’ (RWR) interpretations. To address the affinities and differences between QT and QLTs, the article introduces two new principles: ‘the unambiguity principle’, equally applicable in QT and QLTs, or in mathematics and science in general, and ‘the free will principle’, only applicable in QLTs and not in QT. The article also reflects on the limits of quantum-like sciences (QLSs) and mathematical–experimental science in general in dealing with human thinking and decision making.

This article is part of the theme issue ‘Quantum theory and topology in models of decision making (Part 1)’.

## 1. Introduction

This article offers a reverse perspective on quantum theory (QT) and quantum-like theories (QLTs), theories using mathematical models based on the formalism of QT, in human decision sciences. The article argues that QT, in either of its two currently standard forms (the only ones to be considered here, for either discrete or continuous variables), quantum mechanics (QM) or quantum field theory (QFT), is no longer a theory of the behaviour, in particular motion, of physical objects, as was modern—mathematical–experimental—physics, such as classical physics and relativity, that preceded QT. Instead, QT is a form of decision theory, involving a special nature or, as I shall call it, ‘topology’ of decisions. I use the term topology in part metaphorically in referring to a ‘space’ of possible decisions, but only in part, because it also applies in its proper mathematical sense to the formalism of QT [1,2, pp. 927–930]. This topology includes the concept of free will, which, however, requires qualifications, compelling me to ground it in the concept of decision, which re-delimits that of free will. At least, this character of QT emerges in the present and related interpretations of QT, designated here as ‘reality without realism’ (RWR) interpretations. These interpretations place the ultimate reality responsible for quantum phenomena beyond representation or even conception. I shall focus on QM, the main source of QLTs (which only occasionally use QFT), although my argument concerning QM would apply to QFT. Alternative quantum theories, such as Bohmian mechanics, or alternative interpretations of QM will only be mentioned in passing.

I offer some terminological clarifications first. Physics, as a science, including quantum physics (QP), involves both establishing physical phenomena, in which case one refers to experimental physics, and investigating these phenomena by theoretical means, in which case one refers to theoretical physics. Both relativity theory, special and general, and QM and QFT, are parts of theoretical physics. ‘Classical physics’ also commonly refers to classical physical theories, such as classical mechanics, classical statistical physics, chaos theory or classical electromagnetic theory. I shall retain this use. By QLTs, I refer to theories that use mathematical models based on the formalism of QT beyond physics, specifically in human decision sciences which may adopt QLTs, thus becoming quantum-like sciences (QLSs). Technically, a different interpretation of a theory forms a different theory, because an interpretation may involve concepts not shared by other interpretations. For simplicity, however, I shall refer to different interpretations of QM or QFT, or QLTs. In the case of classical physics and relativity theory, I shall, as is common, refer to theories themselves. The reason is that most interpretations of these theories, including the ones assumed here, are realist or ontological, insofar as the reality responsible for the phenomena considered in these theories is (ideally) represented by them.^1^ By contrast, the proliferation of different and sometimes incompatible interpretations of QM and QFT has been massive, and debates concerning these theories and their interpretations have continued with an undiminished intensity for a century now.

RWR interpretations of QM or QFT and a view of it as a decision science offer a way, one way, to counter the apparent ambiguity in referring to the *ultimate* reality responsible for quantum phenomena, as an independent physical reality. Quantum phenomena themselves can be referred to unambiguously, as, in the present interpretation, following N. Bohr, represented by classical physics with the addition of special relativity in high-energy regimes.^2^ The ambiguity in question amounts to the impossibility of assigning any definitive properties to the ultimate reality responsible for quantum phenomena. This is the meaning of ambiguity assumed here, rather than its common meaning as something inexact. In adopting an RWR interpretation, Bohr, spoke, giving ‘the essential ambiguity’ this sense, of ‘the essential ambiguity involved in a reference to physical attributes of objects when dealing with phenomena where no sharp distinction can be made between the behaviour of the objects themselves and their interaction with the measuring instruments’ [5, v. 2, p. 64]. While this ambiguity had shadowed QP from its emergence with M. Planck’s black body radiation law in 1900, its potential unavoidability became amplified and especially troubling to many physicists and philosophers, most famously A. Einstein, with the introduction of QM in 1925.

I shall also argue that, by considering human subjects as objects of investigation, QLTs confront a much greater degree of ambiguity than QT, which only involves decisions and the free will of the agents conducting investigations. Thus, while retaining most key features of QT, QLTs shift the centre stage of decision vis-à-vis QP. In QP, only the agents make decisions, while the objects under investigation, which are physical entities, make no decisions and have no free will, or are capable of thinking, in the first place. In human decision sciences, the objects under investigation are human subjects, and their decisions and free will take the centre stage, although the decisions and free will of the agents still play the same role in QLSs as in QP. This asymmetry leads to the free will principle (defined below) in QLTs, correlative to the higher degree of ambiguity QLSs confront, vis-à-vis QP. This degree of ambiguity may require theories beyond QLSs and may limit a mathematical–experimental or any scientific handling of human decision making.

Of course, the (RWR) view of the ultimate reality responsible for the phenomena considered as precluding an unambiguous reference to this reality is only acceptable if a theory or interpretation that assumes this view avoids ambiguity in *actual practice* of this theory. RWR interpretations of QM or QFT, or, as I argue here, QLTs, do allow one to do so. Establishing the unambiguous character of QT was one of the reasons for the introduction of RWR interpretations, beginning with W. Heisenberg’s discovery of QM and, more rigorously, Bohr’s work. Although neither used the term RWR or spoke of QM as a decision science, these conceptions were implicit in their views of QM. The absence of ambiguity in defining and communicating all theoretical propositions or experimental findings considered is essential to scientific practice [6, p. 699], here defined as ‘the unambiguity principle’. It dictates *the necessity of unambiguous definitions and communication, at least as unambiguous as possible, of the concepts and propositions (which are also needed to define and communicate concepts) and hence theories or experimental practices in mathematics and science*.

The unambiguity principle allows one to maintain ‘the free will principle’, which is part of the topology of decision in QLTs, although it limits the scope of the latter principle. On the other hand, the free will principle is not applicable in QP, even though the origin of the principle is the Conway–Kochen ‘free will’ theorem [7], which in its original formulation, attributed, as I shall explain, unnecessarily, a free will to quantum objects. The free will principle states: *if one has free will in the sense that one’s decision of the question to ask is not entirely determined by the past, then, some of the respondents must also possess free will to give an unexpected (even for them) answer, instantly change the answer, or not to answer, without this response being predetermined by the past, either that of the respondent or that of the agent*. Admittedly, free will is a vast subject debated throughout Western intellectual history, from the pre-Socratics on (e.g. [8] for a survey). It can only be considered here within a limited scope, defined by the free will principle.

An individual human experience, which defines human decisions and free will, is ultimately singular, even in mathematics or science, and is incommunicable in its entirety. Mathematics and science need to reduce the ambiguity (to which this singularness may give rise) in their functioning as disciplinary fields, although this ambiguity cannot be avoided in individual experience of mathematicians and scientists. By contrast, literature and art embody the uniqueness and ambiguities of human experience in creating and experiencing the works of literature or art. Language and other human means of expression allow for both ambiguity and unambiguity. Mathematics and theoretical sciences are unambiguous not because they are based on something exterior to general experience, thinking and language, but because they capitalize on the capacity of these basic human attributes to make meaning and communication unambiguous and in this sense *objective*. This need not imply the objectivity in the (realist) sense of representing the ‘object’ considered, material in science or mental in mathematics, given the possibility of RWR interpretations, which preclude their representation. They do, however, allow for objectivity in the sense of the unambiguous meaning and communication, in conformity with the unambiguity principle.

I should like, by way of a prologue, to consider the case of a reduction of ambiguity in the concept of a Riemann surface, which played a key role in introducing topology as a new mathematical discipline. This concept was part of the genealogy of Bohr’s concept of complementarity as a parallel form of the reduction of ambiguity in QP. Bohr’s early interests (before physics) were in the philosophy of psychology, and the concept of a Riemann surface attracted his attention in this connection, as discussed in [3, pp. 202–203]. A Riemann surface allows one to remove ambiguity is considering functions of complex variables, such as *ƒ*(*z*) = √*z*. The meaning of *f* (*z*) is ambiguous and hence not properly definable, when considered on the complex plane, because √*z* has two meanings there; √*z* is, however, well defined on the corresponding Riemann surface, because it has a single meaning on each of its two separate sheets. Bohr reflected on this part of the genealogy of complementarity, in his final interview in 1962, shortly before his death:

At that time I really thought to write something about philosophy, and that was about this analogy with multivalued functions. I felt that the various problems in psychology—which were called big philosophical problems, of the free will and such things—that one could really reduce them when one considered how one really went about them, and that was done on the analogy to multivalued functions. If you have square root of *x*, then you have two values. If you have a logarithm, you have even more. And the point is that if you try to say you have now two values, let us say of square root, then you can walk around in the plane, because, if you are in one point, you take one value, and there will be at the next point a value which is very far from it and one which is very close to it. If you, therefore, work in a continuous way, then you—I am saying this a little badly, but it does not matter—then you can connect the value of such a function in a continuous way. But then it depends what you do. If in these functions, as the logarithm or the square root, they have a singular value at the origin, then if you go round from one point and go in a closed orbit and it does not go round the origin, you come back to the same (value). That is, of course, the discovery of Cauchy. But when you go round the origin, then you come over to the other (value of the) function, and that is then a very nice way to do it, as Dirichlet (Riemann), of having a surface in several sheets and connect them in such a way that you just have the different values of the function on the different sheets. And the nice thing about it is that you use one word for the function, *f(z*). Now, the point is, what is the analogy? The analogy is that you say that the idea of yourself is singular in our consciousness—do you think it works; am I doing it sufficiently loud? Then you find—now it is really a formal way—that if you bring this idea in, then you leave a definite level of objectivity or subjectivity. For instance, when you have to do with the logarithm, then you can go around; you can change the function as much as you like; you can change it by 2π, when you go one time round a singular point. But then you surely, in order to have it properly and be able to draw conclusions from it, will have to go all the way back again in order to be sure that the point is what you started on.—Now I am saying it a little badly, but I will go on.—That is then the general scheme, and I felt so strongly that it was illuminating for the question of the free will, because if you go round, you speak about something else, unless you go really back again (the way you came). That was the general scheme, you see. [9, session 5]

There are important differences between these concepts. In the case of a Riemann surface, we only have two or more mutually exclusive mathematical domains in which the function in question is unambiguously defined. In QP, each complementary situation exhibits a different *possible* ultimate reality responsible for each quantum phenomenon, even if they are associated with the same quantum object. To enact both possibilities, two separate measurements on two different quantum objects of the same type (say, two electrons) are necessary. QM predicts this behaviour in two mathematically different ways, for example, by using two different forms of Schrödinger’s equation (for the position and for the momentum, respectively). These predictions are probabilistic, which is not a feature of Riemann’s concept either. Nevertheless, the role of new mathematics in resolving the ambiguity of applying a previously established concept is instructive. What achieves this resolution is a new mathematical structure, extending to a whole new discipline of topology, in the rise of which the idea of a Riemann surface was a key event. Complementarity similarly resolves the ambiguity of simultaneously applying both values of conjugate variables, such as position and momentum, to quantum objects. These values only unambiguously apply in a mutually exclusive way to what is observed in measuring instruments as effects of their interactions with quantum objects. While conceptual rather than mathematical, complementarity is connected to the mathematics of QT, necessary to predict the outcome of possible measurements for either variable on the basis of an already performed measurement of it. These two measurements are mutually exclusive and require incompatible experimental arrangements. On the other hand, one can decide and thus has a degree of free will (to which Bohr refers above), to perform one experiment or another. Complementarity is defined by both the mutual exclusivity of these two situations and the possibility of, by decision, establishing either at any moment in time. In RWR interpretations, it is not possible to apply even individual, rather than only joint, properties to quantum objects. It is possible, however, to apply either complementary property, but never both together, to what is observed, classically, in measuring instruments.

The concept of RWR has been introduced by this author previously and discussed in several works, most comprehensively in [3]. RWR interpretations place the ultimate reality responsible for quantum phenomena either beyond representation, in which case I refer to *weak* RWR interpretations, or even beyond conception, in which case I refer to *strong* RWR interpretations, such as the one adopted in this article. In RWR interpretations, while QM or QFT probabilistically predicts what is observed as quantum phenomena (no other predictions are in general possible on experimental grounds), it offers no representation or even conception of how quantum phenomena come about, thus, precluding one from referring unambiguously to the properties of the ultimate reality responsible for these phenomena. Nor does it describe these phenomena in the present interpretation. These observable parts of these instruments and thus quantum phenomena are represented by classical physics, with the addition of special relativity in high-energy regimes. This assumption (not required for an RWR interpretation) was introduced by Bohr and was made in all versions of his interpretation, including its ultimate, strong RWR, version.^3^ I only claim a logical consistency of RWR interpretations and their accord with the experimental evidence available thus far. Other interpretations of QM (or QFT), or alternative quantum theories, are possible.

In the present interpretation, the equations of QM, such as Schrödinger’s equation, or QFT, such as Dirac’s equation, are *not equations of motions*. They are *equations of decisions (concerning which experiment to perform) and transition probabilities between quantum events*. Accordingly, the reverse symmetry of these equations with respect to parameter *t* in them, which parameter should not be simply identified with time [10], does not affect the sequences of the events considered, irreversibly, proceeding from the past to the future. This character of the equations of QT naturally connects QT and QLTs (in RWR interpretations of QLTs also assumed here) because QLTs have no relation to motion in the way physical theories do. QLTs do of course deal with temporal changes and events in time. Just as it does in the case of QP, the present view of QLSs implies the arrow of time or, my preferred term, the arrow of events from the past to the present to the future, and the corresponding probabilistic view of causality, explained below.

It is the conjunction of these features—complementarity; the character of the equations of QT as equations of decision and transitions probabilities between events, rather than equations of motion; probabilistic causality, and the arrow of events—that defines both QT and QLTs in the present view of both, and thus L in QLT (or QLS), and the corresponding topology of decision in them. This topology is a form of *doubling* of both the reality considered and how one relates to this reality, including mathematically, in which case the term ‘topology’ may also be understood in its properly mathematical sense in connection to the formalism of QT or QLTs.^4^ This doubling is defined by which of the two possible decisions, allowing for a free or sufficiently free choice, made in complementary set-ups. In QT, this doubling allows one to avoid the ontological ambiguity of referring to the ultimate reality responsible for quantum phenomena. The situation acquires further complexity in QLSs, which brings with it a new form of ambiguity, because the objects under investigation are human subjects making decisions, rather than inanimate physical objects, which do not make decisions or have free will. This difference is reflected in the free will principle, only applicable in QLSs and not in QP, while the unambiguity principle applies in both.

## 2. The unambiguity principle and QT as a decision theory

As are concepts of reality in realist theories, the concept of RWR is based in more general concepts of reality and existence, which are assumed here to be primitive concepts and are not given analytical definitions. By ‘reality’ I refer to that which is assumed to exist, without making any claims concerning the character of this existence, claims that define realism, which, in mathematics, is sometimes referred to as a mathematical Platonism. Realist physical theories or realist interpretations of QM or QFT aim to offer at least a conception but more commonly a representation of this reality, usually in terms of the objects considered by a theory. A realist theory would also aim to predict the future course of this reality by means of this representation, either ideally exactly, as in classical mechanics of individual or simple systems, or relativity, or probabilistically, as in classical statistical physics or chaos theory. By contrast, the absence of such claims allows one to place the reality considered or a stratum of this reality beyond representation or even conception. It is this placement that defines this reality or its stratum as an ‘RWR’ and the corresponding interpretation of quantum phenomena and QM, or QFT, as an RWR interpretation. I understand existence as a capacity to have effects on the world. The world may be defined, following L. Wittgenstein, as ‘all that is the case, …the totality of facts, not of things’, and, thus, is real in our experience, individual or shared [11, p. 24]. The universe will be understood here as ‘all that is the case, … the totality of facts’ in physics. A rigorous assumption that something is real can only be made on the basis its effects, such as observed quantum phenomena, assumed here to be represented by classical physics. Classical physics, however, cannot predict these effects, doing which requires a QT, such as QM or QFT. Whether they assume or not that quantum phenomena are represented by classical physics, RWR interpretations place the ultimate reality responsible for these phenomena, as effects of the interactions between this reality and measuring instruments, beyond representation or conception. RWR interpretations do not assume a uniform or unified character of this reality, a character only manifesting itself differently in each experiment. This assumption is in conflict at least with strong RWR interpretations, which preclude any conception of this reality and, hence, that of its unity or oneness.^5^ While each time unthinkable, an RWR-type reality is each time unique, manifesting its uniqueness, in each encounter with this reality, as an effect, each time unique in turn, of this encounter.

It is true that any conception of how anything exists, or even that it exists, including as independent of thought, belongs to thought. It need not follow, however, that something which such concepts represent, or to which they relate otherwise than by representing it, possibly placing it beyond representation or even conception, does not exist. This point was made in 1905 by H. Lebesgue, one of the founders of modern integration and measure theory, in responding to the paradoxes of G. Cantor’s set theory, shaking the foundations of mathematics then. Lebesgue argued that the fact that we cannot define or imagine objects, such as ‘sets’, that are neither finite nor infinite, or neither continuous nor discontinuous, does not mean that such objects do not exist [12, pp. 261–273, 13, p. 258]. Lebesgue did not specify in what domain, material or mental, such entities might exist, but, arguably, had both domains in mind. Admittedly, it is equally impossible to be certain that such a reality does exist. The possibility of its existence, however, reflects both potential limits of human thought and yet its capacity to conceive of this possibility.

Assuming such a reality is a philosophical wager. It was, however, this wager that led Heisenberg to his invention of QM and its mathematics, never previously used in physics. While, as infinite-dimensional over C , this mathematics was continuous (although it contained some discrete algebra), this mathematics enabled QM to predict quantum phenomena, irreducibly discrete relative to each other, with no continuous connections between them assumed by Heisenberg. These predictions were and could have only been probabilistic, which, as noted, is strictly in accord with quantum experiments and was the starting point of Heisenberg’s approach, because these probabilities could not be predicted by classical physics or, in all cases, by the preceding (‘old’) QT. By the same token, the nature of quantum probability is different from that of classical physics, like classical statistical physics or chaos theory. QM, in RWR interpretations, beginning with that (implicitly) assumed by Heisenberg, change the nature of causality and, hence, probability in QT, inviting an introduction of the concept of ‘quantum causality’ versus ‘classical causality’ governing classical physics or relativity. By classical causality, I refer to the claim that the state, X, of a physical system is determined, in accordance with a law, at all future moments of time once its state, A, is determined at a given moment of time, and state *A* is determined by the same law by any of the system’s previous states. This assumption implies a concept of reality, making classical causality ontological, which is precluded by strong RWR interpretations, because the ultimate reality responsible for quantum phenomena is beyond conception. Some, beginning with P. S. Laplace, have used ‘determinism’ to designate classical causality. I use determinism as an epistemological category referring to the possibility of predicting the outcomes of classically causal processes ideally exactly. In classical mechanics, when dealing with individual or small systems, both concepts are coextensive. By contrast, classical statistical mechanics or chaos theory are classically causal but not deterministic because of the mechanical complexity of the systems considered, which limits one to statistical predictions concerning their behaviour.

The reason for my choice of ‘classical causality’, rather than, as is more common, just causality, is the possibility of alternative probabilistic concepts of causality, applicable in QM in RWR interpretations, where classical causality does not apply, one of which is that of ‘quantum causality’. This concept is discussed in detail in [3, pp. 207–218]. For the present purposes, it suffices to state that, while preserving a form of causal influence between events and an arrow of events, from the past to the future, this concept reflects the fact that RWR interpretations change the nature of probability in QP versus that in classical physics. There, the recourse to probability, when necessary, is a practical, epistemological matter, owing to our lack of knowledge concerning the underlying behaviour of the complex systems considered, while their elementary constituents could in principle be predicted exactly. In QT, in RWR interpretations, this recourse arises because there is no knowledge or even conception concerning the behaviour of quantum objects, even the simplest possible ones, such as those associated with elementary particles. Hence, no form of classical causality, which is an ontological conception, can apply. According to Bohr:

[I]t is most important to realize that the recourse to probability [in QP] is essentially different in aim from the familiar application of statistical considerations as practical means of accounting for the properties of mechanical systems of great structural complexity. In fact, in quantum physics we are presented not with intricacies of this kind, but with the inability of the classical frame of concepts to comprise the peculiar feature of indivisibility, or ‘individuality,’ characterizing the elementary processes. [5. v. 2, p. 34]

The ‘indivisibility’ refers to the indivisibility of phenomena in Bohr’s sense, defined, in Bohr’s or other RWR interpretations. ‘Individuality’ refers to the assumption that each quantum phenomenon is unrepeatable, unique. It is the outcome of a unique act of creation, defined by the decision and possibly free will of the agent performing the experiment that gives rise to this phenomenon, thus defining reality at this moment in time, as opposed to locating an already established reality as an observation does in classical physics or relativity. On the other hand, how quantum phenomena come about is beyond the ability of the classical frame of concepts, and ultimately any concepts, to comprise. This situation makes quantum probability not merely a practical matter of dealing with mechanical systems that are classically causal but too complex for tracking and thus predicting their behaviour (ideally) exactly. QP is probabilistic even in dealing with the simplest quantum systems, such as elementary particles, the impossibility manifested in the uncertainty relations. This kind of randomness is not found in classical physics. This is because even when one must use probability there, one deals with underlying individual processes that are classically causal, and in the first place, allow for a realist representation. There are situations, such as those of the Einstein–Podolsky–Rosen (EPR) type of experiments where predictions concerning certain variables are ideally possible with probability equal to one. In view of the specific conditional character of these predictions, however, they do not entail classical causality [3, pp. 207–218]. While quantum phenomena contain this randomness, they are not strictly random, but instead exhibit statistical correlations in certain circumstances, such as in EPR-type experiments. That the intrinsic randomness of individual quantum events coexists with the (statistical) order of quantum correlations is one of the greatest and most famous ‘mysteries’ of quantum phenomena, supporting RWR interpretations.

Historically, the present argument originates in Heisenberg’s thinking that led him to his discovery of QM. I have considered Heisenberg’s derivation of QM in detail previously [3, pp. 101–127], and shall only comment on its aspects pertinent to my argument for QM as a decision theory. Shortly before completing his paper introducing QM [14], Heisenberg wrote to R. Kronig:

What I really like in this scheme [of QM] is that one can really reduce *all interactions* between atoms and the external world ... to transition probabilities. (Heisenberg, Letter to, 5 June 1925; cited in [15, v.2, p. 242])

This statement is telling in reflecting the revolutionary nature of Heisenberg’s thinking. Heisenberg used mathematics never previously used in physics, that of unbounded infinite-dimensional matrices (in effect Hilbert-space operators) over C, replacing the functions of the real variables in classical mechanics. He was famously unaware of the existence of matrix algebra and reinvented it. The non-commutative nature of his variables, standard in matrix algebra, was one of the unexpected and implicative new features of Heisenberg’s formalism, for example, having *PQ − QP ≠ 0*, for the variables associated, respectively, with momentum *p* and coordinate *q*, probabilistically predictable as actual measurable physical quantities. This non-commutativity was related to, and is often interpreted as, the fact that if one performs two such measurements in sequence, the outcomes will be different depending on the order in which they are performed. One needs additional rules, such as Born’s rule, a form of complex conjugation (always giving one a real quantity), allowing one to move from complex to real quantities necessary for defining the probabilities in question. Heisenberg used a limited form of Born’s rule (which is general) applied to the transitions between stationary states of electrons, at which they had constant energy.

Heisenberg adopted a weak RWR interpretations, because, in Bohr’s words at the time, ‘[Heisenberg’s] quantum mechanics [did] not deal with a space–time description of the motion of atomic particles’ [5, v.1, p. 48], rather than in principle excluded a description or conception of how quantum phenomena came about. Bohr’s strong RWR interpretation was introduced a decade later, shaped by his intervening debate with Einstein. In presenting this interpretation in print for the first time in 1937, Bohr spoke of ‘our not being any longer in a position to speak of the autonomous behaviour of a physical object, owing to the unavoidable interaction between the object and the measuring instrument’ [16, p. 87]. If one could form a concept of this behaviour, one would presumably be able to say something about it, unless one uses a concept inexpressible in any language. Bohr never mentioned this esoteric possibility. He clearly held a strong RWR interpretation by then, as confirmed by his other statements, such as, responding to Einstein’s scepticism: ‘In quantum mechanics, we are not dealing with an arbitrary renunciation of a more detailed analysis of atomic phenomena, but with a recognition that such an analysis is *in principle* excluded’ [5, v.2, p. 62].

In arguing for the *unavoidable* interactions between the object and the measuring instrument in QP Bohr was grounding Heisenberg’s view that QM was only dealing with ‘the interactions between atoms and the external world’. QM was only predicting the effects of these interactions, without *necessarily* representing how these effects are possible, thus allowing one to assume that the ultimate reality responsible for these effects is beyond representation or conception. The probabilistic nature of quantum predictions is automatic under this assumption, because in the absence of such a representation, let alone conception, there is no mechanism available for exact predictions. The transition probabilities in question only concern quantum phenomena, technically, the data observable in them. These data are the only observable physical properties versus those of quantum objects. The latter and hence their properties are not observable, regardless of interpretation and are unrepresentable or even unconceivable in RWR interpretations.

As stated, Bohr made an additional assumption, adopted here as well, that quantum phenomena and the data contained in them, along with the observable parts of the measuring instruments, are represented by classical physics, ‘however far the phenomena transcend the scope of classical physical explanations’ [5, v.2, p. 39]. These instruments are also assumed to contain a quantum stratum through which they interact with quantum objects, for no interaction with quantum objects would be possible otherwise. As quantum, this stratum and this interaction are, in RWR interpretations, beyond representation or conception as well. In these interpretations, QP transforms the nature of observation and measurement as understood in the preceding history of mathematics and physics, from ancient Greek geometry, geo-*metry*, to classical physics or relativity, as measuring pre-existing properties of the objects considered. This procedure is replaced by a two-act sequence. The first is the establishment of a quantum phenomenon through the interactions between the quantum object considered and the observational instrument used, making an observation an act of creation, unique each time. Then the data obtained in this act are measured classically.

Establishing quantum phenomena requires special instruments. Human bodies are sufficient in some cases and are good models for the instruments used in classical physics, which similarly do not disturb what is observed. This is not the case in QP. Relativity also requires instruments, rods and clocks, because relativistic effects cannot be perceived by human bodies. This dependence, however, still allows relativity to be a classically causal (in fact deterministic) and in the first place, realist theory, because one can still observe the systems considered without sufficiently disturbing them. The difference between objects and phenomena is still in place in classical physics and relativity, in accord with I. Kant’s view of objects as things-in-themselves versus phenomena as appearance to our mind. That means that one still ultimately deals with phenomena and not objects. The fact, however, that one can neglect or control the interference of observational devices into the course of the phenomena allows one to treat objects and phenomena as the same and the behaviour of objects as independent for all practical purposes in either theory. This is no longer possible in QP, regardless of interpretation, because quantum objects cannot be observed independently or treated as so observed. Nobody has ever seen an electron or photon, or any quantum object. One can only observe traces of their interactions with observational instruments, while the ultimate origin of these traces is beyond the reach of our instruments and, in strong RWR interpretations, beyond the reach of thought. This is a more radical view than that of Kant. While things-in-themselves were assumed by Kant to be beyond representation or knowledge, they were not assumed to be beyond conception, even if such a conception could only be practically justified by its workable applications [17, p. 115]. In strong RWR interpretations, what is practically justified is not a possible conception of the ultimate reality responsible for quantum phenomena, but the impossibility of such a conception. Around 1937, as part of his ultimate interpretation, Bohr introduced his concept of ‘phenomenon,’ extending and yet transcending Kant’s view:

I advocated the application of the word phenomenon exclusively to refer to *the observations obtained under specified circumstances*, including an account of the whole experimental arrangement. In such terminology, the observational problem is free of any special intricacy since, in actual experiments, all observations are expressed by unambiguous statements referring, for instance, to the registration of the point at which an electron arrives at a photographic plate. Moreover, speaking in such a way is just suited to emphasize that the appropriate physical interpretation of the symbolic quantum-mechanical formalism amounts only to predictions, of determinate or statistical character, pertaining to individual phenomena appearing under conditions defined by classical physical concepts [describing the observable parts of measuring instruments]. [5, v.2, p. 64]

As defined by ‘*the observations* [already] *obtained under specified circumstances*’, phenomena strictly refer to events that have already occurred and not to possible future events, such as those predicted by QM. This is the case even if these predictions are ideally exact, which, as noted, they may be in certain circumstances, such as in EPR-type experiments. The reason that such a prediction cannot define a quantum phenomenon is that a prediction for variable *Q* (related to a coordinate *q*) cannot, in general, be assumed to be confirmed by a future measurement. This is because one can decide to perform a complementary measurement, that of *p* (the momentum), which will leave any value predicted by using *Q* entirely undetermined by the uncertainty relations and thus irrevocably preclude associating a physical reality corresponding to *q* [3, pp. 210–212]. This possibility, which manifests human decision and free will, makes EPR-type predictions conditional. This point has major implications for understanding the EPR experiment and for countering EPR’s argument along the lines of Bohr’s reply [3, pp. 227–257]. One can never speak of both variables as having an exact value unambiguously and hence properly define both, even if they are associated with measuring instruments. No reference even to a single property of a quantum object, is possible at all in RWR interpretations even at the time of observation, let alone independently. On one hand, there is always a discrimination between an object and an instrument, and on the other, the impossibility of physically separating them, making the object inaccessible. By contrast, a reference to what is observed can be unambiguous and communicated as such, and in this sense is objective.

In departure from classical probability theory, the probabilities involved are, in general, non-additive, or non-Kolmogorovian: the joint probability of two or more mutually exclusive alternatives in which an event might occur is not *always* equal to the sum of the probabilities for each alternative, as in Kolmogorovian probability theory, where p(A) + p(Ā) = 1 (Ā is an event independent of A, and p is probability). In QM, while there are *compatible* events in which this Kolmogorovian law applies, there are, as in the case of complementarity, also mutually exclusive, incompatible events, to predicting which this law does not apply. To accommodate such cases, all quantum-mechanical probabilities are derived by using the addition of ‘amplitudes’ and Born’s rule. The term ‘amplitude’ is adopted from classical wave theory because the equations of QM or QFT contain entities formally analogous to amplitudes in classical wave equations. These amplitudes, however, no longer serve their classical function as part of a continuous representation of wave motion. They are abstract mathematical entities, ‘probability (density) amplitudes’, related to possible alternatives in which a future event might happen. Born’s rule is applied to the sum of these amplitudes, giving real numbers, which sum, suitably normalized, is equal to the sum of probabilities of these events. This sum is, however, not always equal to the sum of probabilities of each as independent events, because of the incompatible cases, where it is not.

As Bohr emphasized, in spite or even because of their radical epistemological nature, his and, by implications, other RWR interpretations remain consistent with ‘the basic principles of science’ [6, p. 699]. That includes ‘the unambiguous logical representation of relations between experiences’ and hence the possibility of unambiguously communicating the scientifically relevant contents of these experiences, in sum the unambiguity principle [5, v.2, p. 68]. It is not coincidental that Bohr refers to *experiences* rather than experiments. It is ultimately our experiences that make science, or mathematics, possible. We are, however, also able to establish them as science and mathematics as forms of disciplinary practice. In science, or mathematics, experiences combine individual (and thus partly subjective) aspects with shared (more objective) ones, with the latter defined by the unambiguity of communication. Mathematics and science must, however, maximally reduce the ambiguity of defining and communicating their findings. This was the definition of ‘objectivity’ in Bohr, versus realism, where ‘objectivity’ refers the representation of the ultimate reality responsible for physical phenomena as an independent reality, thus assumed to be representable, an assumption ‘*in principle* excluded’ in RWR interpretations of QM [5, v. 2, p. 62]. A representation and thus realism is possible and, in Bohr or the present interpretation is assumed, in considering quantum phenomena, represented by classical physics. (It is, again, possible to have an RWR interpretation without this assumption.) The mathematical formalism of QM or QFT can also be unambiguously communicated, as can be any mathematics. Bohr comments on mathematics and its ‘special role in physics’ in this connection on the same occasion: ‘it may be stressed that, just by avoiding the reference to conscious subject which infiltrates daily language, the use of mathematical symbols secures the unambiguity of definition required for objective description’ [5, v.2, p. 68].

The uncertainty relations exemplify the quantum-mechanical situation here outlined, including the unambiguity principle. An uncertainty relation is represented by a formula Δ*q*Δ*p* ≅ *h*, where *q* is the coordinate, *p* is the momentum in the corresponding direction, which are *measurable quantities* observed in instruments, *h* is the Planck constant and Δ is the standard deviation. There is nothing unambiguous about the formula. It represents an experimentally confirmed law independent of any theory. An interpretation of the uncertainty relations is a subtle issue. They do, however, imply that the simultaneous *exact* measurement of both variables is not possible. On the other hand, it is possible to measure each ideally exactly. For Bohr and here, the uncertainty relations mean that one cannot even define both variables simultaneously, while one can define either at any moment in time.

This situation is a manifestation of Bohr’s complementarity. Complementarity entails

(A) a mutual exclusivity of phenomena (or possibly other entities, such as concepts); and yet
(B) the possibility of considering, by decision, each one of them separately at any given point; and
(C) the necessity of considering all of them at different moments of time for a comprehensive account of the totality of phenomena that one must consider in QP.

As the uncertainty relations, complementarity is not a feature of QT, but of quantum phenomena. QM and QFT are, however, fully in accord with complementarity as they are, correlatively, with the uncertainty relations. Complementarity implies two incompatible cases of what is observed, as quantum phenomena. In complementary situations, the *possible* information concerning a quantum object, defined by the observable effects of the interactions between this object and the instrument used, can only be obtained in mutually exclusive experiments. On the other hand, once obtained, either information, say, that of the position, is the complete *actual* information, as complete as possible, about the object at this moment in time, keeping in mind that the object itself is beyond knowledge or conception. One could never obtain the complementary information (concerning the momentum), because this would require one simultaneously to perform a complementarity experiment on the same object, which is impossible. By (B), however, one can always decide to perform either experiment and has a free will to do so.

Complementarity is thus not only about the mutual exclusivity of the entities considered, but also about performing quantum experiments by human agents. One can set up a device to perform an experiment or treat a natural event, such as a radioactive emission, as an experiment. Any such set-up is only possible by a human agent or by a device, such as a computer, which, at least as things stand now, must be programmed by a human agent to do so. That one has freedom, at least a relative freedom, to decide which experiment to perform is in accordance with the very idea of experiment in all science [6, p. 699]. Contrary to classical physics or relativity, however, implementing our decision in QP allows one to make only certain types of predictions and will irrevocably exclude *complementary* types of predictions. Our decisions establish the reality considered and its possible future course in two alternative ways. (Wave-particle complementarity, with which the concept of complementarity is often associated, can also be seen in this way, but it will be put aside here because it had not played a significant role in Bohr’s argumentation [3, pp. 205–206].)

The role of decision is thus crucial in QP and is one of the key differences between it and classical physics or relativity, which do not contain either the uncertainty relations or complementarity [3, pp. 209–218]. We always have freedom, at least relative freedom, to make or to change our decision, and by doing so define a new reality and its possible future course. This freedom is relative because it may be limited by various factors (such as social, professional or psychological), which makes ‘decision’ a necessary category, alongside that of free will.^6^ Nor, given the *uncontrollable* nature of the interactions between quantum objects and measuring instruments, can one control the outcomes of quantum experiments [6, p. 697]. One can only control the behaviour of observational instruments because they can, in their observable parts, be described classically. Complementarity helps to establish the unambiguous meaning of what can be said about quantum phenomena, while establishing the difference between QP and classical physics or relativity by virtue of a different topology of decision. In classical physics or relativity, all our decisions can be defined in a single domain of the reality considered, where we make predictions, exact or probabilistic, in the same probability space. This is sometimes referred to as the ‘unicity’ of this domain, which may be the Universe as a whole, as in Newton’s *Principia*, where the Universe is governed by absolute space and time, or as in relativistic cosmologies without these concepts. This unicity is manifested in the equations used, such as Lagrangian equations, which enable the representations and predictions concerning all variables considered, such as those of position and momentum, as in classical physics:


$$\frac{\partial L}{\partial q_{j}}-\frac{d}{dt}\frac{\partial L}{\partial {\overset{˙}{q}}_{j}}=0.$$

Relativity only allows one to maintain this kind of unicity locally, in the case of general relativity by means of Lagrangian equations as well, Einstein’s equations.

In QT or QLTs all domains considered, local or global, are doubled by our decisions to establish one interrelated set of them or the other, without the possibility of unifying them, which may be seen as the ultimate meaning of Bohr’s complementarity. The equations of QM or QFT, say, Schrödinger’s equation, can only be written for one physical reality and in one mathematical ‘space’, a Hilbert space over C, rather than being defined over R, as are the equations of classical physics and relativity, a key aspect of the mathematics of QM or QFT versus that of classical physics or relativity. A measurement of the coordinate, at time *t*_1_ allowing one to use one form of Schrödinger’s equation, for the position variable, to predict (using Born’s rule) the probability of a future position measurement, within a given range, at future time *t*_2_:


$$i\hbar \frac{\partial }{\partial t}\psi \left(x,t\right)=\frac{1}{2m}{\left(-i\hbar \frac{\partial }{\partial x}\right)}^{2}\psi \left(x,t\right)+V\left(x,t\right)\psi \left(x,t\right).$$

A measurement of the momentum (in the same direction) at time *t*_1_ would allow one to use a different form of Schrödinger’s equation, that for the momentum variable, to predict the probability of a momentum measurement within a given range at future time *t*_2_:


$$i\hbar \frac{\partial }{\partial t}\phi \left(p,t\right)=\frac{p^{2}}{2m}\phi \left(p,t\right)+V\left(i\hbar \frac{\partial }{\partial p},t\right)\phi \left(p,t\right).$$

These equations can be mathematically transformed into one another by using the Fourier transform:


$$\phi \left(p,t\right)=\int dx\frac{e^{-i\frac{p}{\hbar }x}}{\sqrt{2\pi \hbar }}\psi \left(x,t\right).$$

This doubled architecture cannot, however, be unified into the single physical domain considered or allow for a single equation or a single system of equations that would enable one to handle both variables at any moment in time, as is possible in classical physics or relativity. One or the other complementary decision enables us to establish one reality and one possible future course of reality, while in principle excluding the existence of the complementary reality by doing so. There are compatible, rather than mutually exclusive, predictions (using commuting operators) associated with different variables. In such cases, the topologies of these decisions are unified. A defining feature of QP is, however, that they cannot always be unified, and when they cannot be, they create incompatible actual and possible realities, rather than describe different parts of the same reality. In Bohr’s words:

We are not dealing with an incomplete description characterized by the arbitrary picking out of different elements of physical reality at the cost of [sacrificing] other such elements, but with a rational discrimination between essentially different experimental arrangements and procedures which are suited either for an unambiguous use of the idea of space location, or for a legitimate application of the conservation theorem of momentum. Any remaining appearance of arbitrariness concerns merely our freedom of handling the measuring instruments, characteristic of the very idea of experiment. [6, p. 699]

While applicable in all physics, in QP, this freedom splits the ‘topology’ of possible decisions into two disjoint parts, rather than selecting one or another part of the connected whole.^7^ Each decision defines new reality and a new possible course of reality, rather than merely following the course of a pre-established reality. The words free and freedom, implying the quantum topology of decision, are used six times in Bohr’s (six-page long) reply to EPR, cited here. According to Cantor, ‘The essence of mathematics is its freedom’ [13, pp. 91–93]. So is the essence of science, with mathematics playing a major role in enabling this freedom in mathematical-–experimental sciences, such as physics, sometimes by experimenting with mathematics in them. Heisenberg did so in reinventing matrix algebra able to predict the data observed in quantum phenomena, while divorcing it, along RWR lines, from the ultimately reality responsible for these phenomena. In RWR interpretations, any reference to the independent properties of this reality is ambiguous. Notwithstanding this ambiguity, however, QP, including as a decision science, remains a mathematical–experimental science because of the unambiguous character of the mathematics of QT and the description of quantum experiments by classical physics. Human decision sciences, including QLSs, may, I argue, encounter new difficulties and potential limits in doing so.

## 3. The free will principle and QLTs

I begin with qualifications concerning QLTs and QLSs as considered here.^8^ Although our thinking is commonly assumed by QLTs, and is assumed here, to be due to the neurological workings of the brain, it is not necessary to assume, and is not assumed here, that the aspects of human thinking treated by these theories arise from the quantum physics of the brain. QLTs may apply even if the physics of the brain is classical and may be used, as will be used here, independently of the physics of the brain. There are theories that view consciousness or thinking as an effect of the physics of the brain, such as those by R. Penrose, beginning with [20] (arguing that this physics is beyond QT), or by using QFT, Vitiello [21] and Khrennikov *et al.* [22]. These theories are hypothetical. How the physics of the brain makes thinking and consciousness possible remains an unanswered question, sometimes referred to as ‘the hard problem of consciousness’ [23]. Why not of thinking? This may be because our manifested inner experience is conscious, and our unconscious thinking is *inferred* from this conscious experience. In any event, while assumed to be responsible for all mental processes, the brain will be treated here as, *physically*, a ‘black box’, relating the informational input and output, from either the outside or the inside of a human subject. The workings of this black box will be given a strong RWR interpretation, in parallel with RWR interpretations of QM, where the black box is the ultimate reality responsible for quantum phenomena as effects of the interaction between this reality and observational instruments.

What would then be shared by QT and QLTs is their conceptual structure, entailing the same topology of decision. The key features of this topology, discussed in §2, are transferable to a large class of QLTs, thus allowing for RWR interpretations of them. The greater role of ambiguity in the phenomena considered by QLSs and QLTs complicates this transfer and may limit the effectiveness of QLTs and other, possibly all, mathematical theories of human thinking and decision making. The phenomena considered in QLSs may not allow one to handle them unambiguously. That, in contrast the unambiguity principle, which applies in both QP and QLSs, the free will principle only applies in QLSs, reflects these difficulties.

The main reason for these difficulties is that human sciences dealing with human decision making, including QLSs, consider both the agents of investigation *and* the objects under investigation as *human subjects*, systems composed of consciousness and the unconscious (C–UC). By contrast in QP, when considered as a decision theory, only the agent is considered as such a system. This difference is manifested in the main shared experimental feature of QP and QLSs, the irreducible role of observation in the constitution of the phenomena considered. This role is defined by using observational instruments and the decision by an agent which experiment to perform in QP, and by means of questions asked by agents, thus, interfering into the thinking of human subjects as objects under investigation, in QLSs. This aspect of QP makes it fundamentally different from classical physics or relativity, where the decisions of the agents do not affect the ultimate reality considered. QLSs are dealing with mental rather than physical reality. Human subjects, as the objects of investigation, become the primary decision systems considered. The subject’s decisions, as communicated to the agent, are manifested in the subject’s consciousness, although the subject’s unconscious may shape them, bringing into play the C–UC system of this subject. The agent is, too, a decision system akin to that of the agent in QP. The decisions concerning which experiment to perform are usually made in advance, even when they are made or changed right before the experiment. The quantum-like aspects of the agent’s thinking are disregarded, as are quantum aspects of agential systems in QP.^9^

As in QT, however, only the agent or a theorist using the data can apply a QLT, for example, a wave function, to assess the behaviour of the subjects considered. One cannot say, as some do, that a wave function belongs to the subject’s thinking enabling this subject’s response to the question asked, or that possible alternative answers by the subject to the same question are in a quantum superposition, any more than one can ascribe the wave function or a superposition to a quantum object in QP. A response to the question posed by an agent involves a decision and free will of the subject, which may require quantum-like thinking and is treated as such by the QLT used. This treatment will involve a wave function or a superposition of state vectors, *and not the subject’s possible answers*, as is sometimes claimed as well. These mathematical means allow the agent and only the agent, and not the subject, to estimate the probabilities of alternative responses. Speaking of the superposition of answers is misleading, just as are statements concerning the superposition of the outcomes of quantum experiments. Neither is in superposition. As explained, what is in superposition are probability amplitudes for possible outcomes of quantum events or for possible answers in QLT experiments. These amplitudes, cum Born’s rule, allow the agents but not the subjects considered, to estimate the probabilities of possible outcomes. A QLS is an activity of human agents conducting investigations in the corresponding domain and not an activity of human subjects as objects of investigations. These investigations do, however, concern these subjects’ decision-making, which makes the subjects’ decisions central to QLSs. In QP, there are no decisions by *the objects under investigation*, which, as physical entities, have no capacity for thought and hence for decisions or free will.^10^

In QLTs, one deals with two interactive decision systems, the one, akin to QT, of the agent, which may be treated as classical, and the other is that of the subject (as the object of investigation), which, as a C–UC system, may combine quantum-like and classical strata in generating its responses, although these responses themselves are treated classically, as in QT. One could, for example, see consciousness as performing a kind of ‘measurement’ on the unconscious as unknown reality [26]. One can then see this situation as parallel to that of quantum phenomena and the unknowable and even unthinkable ultimate reality responsible for them [18,19]. One could also experiment with one’s own C–UC system, as S. Freud did and innumerable other psychoanalysts have done since, thus far usually considering both conscious and unconscious processes classically, rather than the unconscious as QL, as discussed in [18,19].^11^ Under either assumption, however, one still functions as an exterior agent considering one’s own thinking as a system under investigation.

Performing an experiment in physics is sometimes seen as posing a question by an agent, for example, the question concerning where a trace of an electron will be observed at a later time *t*_2_ after one measured its position at time *t*_1_. The *question* is to whom such a question is posed and who answers it. One could, *metaphorically*, say that nature or an electron gives an answer, but only metaphorically. In the present view, nature no more answers questions than asks them. Only we do. There have been views of quantum objects as having a free will or possessing consciousness enabling such a response. One such view was famously and even proverbially suggested by Dirac long ago. These arguments have always been controversial and those of Dirac were quickly challenged [3, pp. 94–97]. A more tempered view for a possible consciousness (small in degree) of inanimate nature, including elementary particles, is offered in [28]. There is, however, neither any evidence for assuming any degree of consciousness or thinking in inanimate nature.^12^

In introducing the free will principle, I do, nevertheless, take advantage of a case of attributing quantum objects a free will, in the Conway–Kochen ‘free will’ theorem [7]. In the initial, controversial, formulation of the theorem, free will was equally attributed to both the agents and quantum objects, specifically elementary particles. The theorem states that, given certain additional assumptions (which have to do with locality, spin and entanglement), if we have a free will in the sense that our choices are not a function of the past, then, some elementary particle must also possess a free will. One can, however, express what is at stake in the theorem without any appeal to free will on the part of quantum objects. The theorem could be and has been reformulated as follows. If one assumes (with the same additional assumptions) free will of the agents, then the outcomes of the individual measurements cannot be determined by anything prior to the experiments. This is a manifestation of a more general feature of QP: the outcome of an individual quantum experiment is, in W. Pauli’s phrase, ‘in general not comprehended by laws’, even probabilistic laws [29, p. 32]. Individual quantum events are random, while coexisting, enigmatically, with the statistical order of quantum correlations. The free will *principle* proposed here, by contrast, only applies in QLSs and not in QP. The concept of decision qualifies this principle by limiting the freedom of the will, while still giving it a key role. I restate the principle: *if one has free will in the sense that one’s decision of the question to ask is not entirely determined by the past, then, some of the respondents must also possess free will to give an unexpected (even for them) answer, instantly change the answer, or not to answer, without this response being predetermined by the past, either their own or that of the agent*.^13^

To illustrate the difference between QP and QLSs here outlined, I should like to consider the Clinton–Gore experiment, paradigmatic in QLTs and mentioned in many expositions of them. The experiment, conducted over two decades ago at the time of President Clinton’s sex scandal, is that of asking two questions, ‘Do you generally think President Clinton is honest and trustworthy?’ (which, asked by itself, tended to elicit the negative answer) and ‘Do you generally think Vice President Gore is honest and trustworthy?’ (which, asked by itself, tended to elicit the positive answer) in two sets of trials by reversing their orders, which exhibit two statistically different outcomes. The case was seen as parallel to complementary measurements in QP and was similarly treated by non-commuting operators in the QLT formalism used. I have considered the case previously [30,32] and shall only focus on those aspects of it that relate to the topology of decision and the free will principle not addressed in these earlier works.^14^

First, I recapitulate how complementary situations appear in QP in RWR interpretations, as defined by the decisions of the agents concerning which of the possible complementary measurement, *Mq*, of variable *q*, to perform, which determines what kind of prediction, in general probabilistic, one can make concerning futures measurements, which would test this prediction. Once either measurement is performed, the exact value of the other possible measurement, *Mp,* for the complementary variable *p*, cannot be known, or in present view defined. Hence (versus as will be seen presently, quantum-like experiments) no information from *Mq* can be carried to *Mp* and no inference concerning *p* can in principle be made, because to do so would require the complementary measurement, which cannot be performed, and any preceding measurement of *p* made obsolete by *Mq*. Quantum predictions can only be based on the last observation performed, which is a unique act of creation of quantum phenomena, observed classically, in the instrument used, rather than a measurement of the preexisting property of a quantum object, which remains beyond knowledge or even conception. The non-commutativity of QM reflects this fact, including in the case of two *reversed* complementary measurements. These measurements give us different outcomes by virtue of being such an act, rather than by measuring preexisting values of the properties of quantum objects.^15^ This is the case even in dealing with a single variable, let alone both complementarity variables, which cannot be exactly measured jointly by the uncertainty relations and require two different quantum objects to reverse the sequence of measurements of these variables. In classical physics, these outcomes are the same because, neglecting, as one can, the interference of observational instruments, both variables can, in principle, be determined together at any moment in time. Even if one measures only one, the other is definable, reflecting the fact that one merely decides to know one or the other aspect of *the same reality* unfolding classically causally, rather than defines two incompatible complementary realities, in accord with Bohr’s concept.^16^

Turning to complementarity in QLSs, I note first the basic that one could never be certain either what another person ultimately thinks even if that person provides as much information as possible concerning this thinking. In fact, one cannot be entirely certain about the content of one’s own thinking because of the unconscious. That we know or assume that others think and yet cannot ultimately know what they think is in the nature of human interactions, in which consciousness may also functions as an instrument of concealment. These circumstances have major implications for quantum-like experiments. Thus, both opinions, one concerning Clinton and other Gore, can exist simultaneously in the mind of the subject, while one of them is unknown to the agent, if the agent only asked about the other. The subject may also have a set opinion, positive or negative, concerning Gore (or Clinton) which will not be affected by the question concerning Clinton (or Gore), as the first question in the sequence. The answer to each could be definitive, as either quantum measurement is in a complementary situation in QP. The statistics of the data obtained will, however, be different, even if the number of instances of this type or other fluctuations of affecting the second question by the first in the sequence considered on the second is relatively small in the sample considered in the experiment. In complementary sequential measurements in QP this number is effectively zero, and there is no causal influence, akin to that in the Clinton–Gore experiment, of the first measurement on the outcome of the second. Numerical data in quantum-like experiments may also appreciably differ from sample to sample, while they do not in QP. In addition, in the present interpretation, the simultaneous *definition* rather than only the simultaneous exact measurement of both complementary variables is not possible. By contrast, the subject can, again, have a well-defined simultaneous opinion about Gore, unknown to the agent, to whom only the subject’s opinion about Clinton is communicated. Only one complementary variable can be well-defined, as observed the instrument used, and never the other in QP.^17^

It is, accordingly, not surprising that, while sharing some of the key features with the mathematics of QT, such as Hilbert spaces (over C), non-commutativity and the non-Kolmogorovian nature of probability, a QLT may mathematically differ from that of QT in details, for example, in using different Hilbert spaces or operator algebras than in QT, or different types of measurements than in QT.^18^ There are also differences between QM and QFT, or among different forms of QFT. In all these theories, however, one is dealing with strictly quantum phenomena in all experiments considered. The situation is more complicated in QLSs, because some of the experiments there involve classical strata within the reality responsible for the phenomena considered. In the Clinton–Gore experiment and most quantum-like experiments, the statistics of the outcomes are in part defined by *classically causal* relationships between the two answers, ‘measurements,’ in a given sequence, for well-established psychological reasons. One can expect these answers and (roughly) their statistics in advance because of these classically causal relationships. While experimentally confirmed statistics are important, it would be surprising if one’s view (conscious or unconscious) of Clinton would not affect one’s view of Gore, or vice versa, in most, although not all, cases. In QP, at least in RWR interpretations, there are no classically causal relationships *in any situation*. Once one measures exactly one complementary variable, say, the position, no expectations at all are possible as concerns a future value of the other, the momentum. This is an experimental fact, and it is true even if one assumes classical causality, as in certain (realist) interpretations of QM or in Bohmian mechanics. When one is asked the second question, concerning the momentum, the *information* carried over from the preceding question, concerning the position, *does not and, in principle cannot, influence* the response to this new question, or the future predictions from this point on, as it may in quantum-like experiments, such as, as indicated above, in the Clinton–Gore experiment. Even if there are classically causal influences in QP, as is assumed in some interpretations or theories (such as Bohmian mechanics), they are much more difficult to establish and are debated. There are no debates concerning classically causal psychological relationships in many situations. It is not surprising either that QLTs or QLSs are multiple, as opposed to the single theory, QM or in high-energy regimes, QFT (comprised of separate theories as it may be), predicting quantum phenomena, as reflected in the role of *h* in all of them; *h* is a fundamental constant of nature that enters all quantum measurements and predictions. There is no comparable constant in QLTs.

Although both the unambiguity and the free will principles offer a new perspective on QLSs versus QP, better arguments for QLTs have been attentive to the complexities just outlined and the resulting differences between QT and QLTs. An instructive example is Z. Wang and J. Busemeyer’s argument for ‘reintroducing complementarity in psychology,’ and, by implication, other QLSs. My limits here prevent me from considering their compelling analysis in detail. I should only reference two of their key points, one now and one in closing the article. Thus, they note that ‘the order effects’ in the Clinton–Core experiment could have different degrees: ‘In a sense, in psychology we could understand this as “reduced incompatibility” of the two questions, or the two sets of [projector operators] are “more nearly commutative”, although in QP, the original concept of complementarity does not have the notion of degrees of complementarity (events can be differentiated only by being [strictly] complementary or not)’ [35, p. 2, fig. 2]. In other words, as I have argued here via the free will principle, the situation is not strictly parallel to QP and may require adjustments of the formalism, depending on the case or domain of a decision science.

From the present perspective, what makes the phenomena considered in QLTs most akin to those considered in QT is the essential individuality and ultimately uniqueness of human thinking of each human subject and in each instance of this thinking, in this respect parallel to each quantum phenomena as a unique act of creation [30,32]. This affinity remains *partial* in view of structural differences here considered, especially by introducing an additional ambiguity into this uniqueness, resulting in the free will principle. Nevertheless, it is important, including in reflecting a potential need of one or another form of QLS in human decision sciences. As noted, asking the subject *any question* may be seen as quantum-like interference into the unknown and even ultimately unknowable (to the agent) reality of the subject’s thought. This interference elicits a conscious response, observed by the agent, akin to an observation in quantum experiment, where this interaction is only enabled and always mediated by a technological device. In quantum-like experiments, one only deals with mental technology. This interference defines a future thinking of the subject and the arrow of events, which may be either interior or manifested in the world, for example, by giving an answer to a question. If one asks the question *just about Clinton*, the subject’s thinking *might become* oriented in a particular way, which will shape the subject’s answer, in accordance with this orientation, concerning the subject’s attitude toward Gore, if such a question is asked next. This reorientation is not certain by the free will principle and does not happen in some cases, in which bringing up Clinton has no effect, as explained above. The possibility of this change does, however, affect the statistics of the Clinton–Gore and similar QLT experiments. The same structure is in place if the first question is about Gore, although the specific outcome may be different, because one deals with a situation mutually exclusive with the first one. These two alternative *sequences* may be seen as complementary in accord with Bohr’s concept, because one deals with two mutually exclusive possible courses of reality, rather than representing two different parts of the same reality. While separate answers concerning Clinton or Gore are not necessarily complementary by themselves, the two alternative sequences in question may be viewed as complementary, leading to the statistics obtained, still, as explained, not the same as these statistics would be in QP. These sequences manifest the topology of decision akin to that in QP, but with the free will principle added. In QP, by contrast, two such sequences are complementary because the measurements of the variables involved, such as the position or the momentum, are always complementary, correlatively to the uncertainty relations. There are no real analogies of the uncertainty relations in QLTs, only rough ones, in part because there is no *h*, numerically defining the uncertainty relations. On the other hand, there is no free will principle in QP.

The question becomes how far QLTs or possibly any mathematical theories can reach in dealing with these levels of complexity and ambiguity, which are much greater than in physics, even in QP, including QFT, highly complex mathematically as the latter is. As all modern physics, QP deals strictly with physical reality, even as, in the present view, a form of decision science as concerns the role of the agent of a given investigation.^19^ This role is, for all practical purposes, subtracted by classical physics and relativity, the subtraction known as the Galilean reduction (versus Aristotle’s physics dealing with both nature and thought). The Galilean reduction is, however, twofold, in fact threefold, because the mathematical–experimental nature of modern physics is predicated on the double reduction. The first is defined by dealing strictly with physical reality, and the second by a mathematical idealization that requires disregarding those aspects of nature that cannot be mathematically idealized. There is, however, a third, subtler reduction. It is based on disregarding the unique reality of each individual subject, involved in creation of physical phenomena, and thus considering only the single physical reality of nature. QP changed this by bringing in human agents and, thus, their individual reality and the quantum topology of decision into the practice of QP, while keeping its mathematical–experimental character, including the unambiguity principle, by retaining the Galilean reduction as concerns the objects considered as natural objects.

Retaining this character becomes difficult in dealing with human thinking, which brings with it new levels of ambiguity, reflected in the free will principle, that human decision sciences must reduce, especially if they aim to use mathematical models, classical or QL. It might, accordingly, be difficult to expect an effectiveness of mathematics comparable to that in natural science. As Wang and Busemeyer observe:

[I]t is true that compared to quantum physics, which provides rigorous and precise predictions about physical phenomena, psychological theories involve many more random variables that are hardly controlled, resulting in lower precision in prediction. To be fair, this is a general challenge that could be raised for any theories in the behavioural and social sciences. Through rigorous model comparison, empirical studies have shown that quantum models provide an elegant new way to specify general and vague verbal theories in psychology, and better explain and predict many phenomena puzzling to classical models, leading to highly testable models. [35, p. 4]

I put aside the first part of the claim, including the ‘elegance’ of QLMs, which seems to me secondary. Also, verbal (which I take to mean non-mathematical) needs not be ‘vague’, and non-mathematical theories may be and have been effective in these domains. This is because they can deal with and resolve those more complex ambiguities of human thinking in confronting which mathematical approaches failed thus far or have not yet been applied. In question are the conditions and limits of their effectiveness, including when using QLTs. Given the experimental and theoretical findings of the last half a century, beginning with A. Tversky and D. Kahneman’s pioneering work, one might expect QLTs to be more effective than classical-like ones. Even in simple cases, such as that of the Clinton–Gore experiment, our thinking appears to be capable of decisions that classical-like theories may not be able to handle, although the issue is not entirely settled. There might also be mathematical theories that are more effective in dealing with these phenomena than either classical-like ones or QLTs. It would, however, be difficult to deny that QLTs are at least likely to better predict many phenomena in human decision-making than classical-like theories. A long list of works would support this view, such as, to mention some influential ones [34,36–42].

The main question of interest in this article is why QLTs may be more effective. I have argued that there are deeper conceptual reasons for this effectiveness, defined by the topology of decision (shared with QT) and its implications, underlying the mathematics of QLTs. On the other hand, the complexities, including greater ambiguities and randomness, which, as manifested in the free will principle, define human subjects as the objects of investigation in QLSs versus material objects of investigation in QP, make one confront a situation that appears to exceed what even the agential topology of decision can handle. These complexities are difficult and perhaps ultimately impossible to treat by scientific means, especially mathematically, as required by the unambiguity principle. The question, then, is how far, given the free will principle, one could in principle extend the effectiveness of QLTs, possibly modelled of QFT, or even any scientific theories, insofar as they are defined by the unambiguity principle. This question makes the situation akin to that in fundamental physics now, in which a new theory is required to bring together gravity, now handled by general relativity, and other forces of nature, now handled by QFT. While there is no theory enable to do this, the current hypothetical proposals to achieve this aim, such as string or M-brane theory, indicate that the mathematics likely to do so is bound to be extraordinarily complex [43]. It would be surprising if extending QLTs to more complex human thinking or expression would be anything less complex mathematically. It is also possible that the complexity and ambiguity of human thinking will ultimately be beyond the reach of any mathematical–experimental theories. Such limits may even exist in fundamental physics, as was surmised by R. Feynman because of the unresolved complexities of QFT, without giving this supposition much of a chance to be realized, which indeed appears unlikely [44, pp. 57–58].

This complexity of human thinking and decision-making are suggested by T. S. Eliot’s famous lines:

In a minute there is time

For decisions and revisions which a minute will reverse.

(‘The Love Song of J. Alfred Prufrock,’1915, ll.47–48)

Our experience may contain too many interconnective trajectories and changes in them, involving endlessly multiplying and fast ‘decisions and revisions’, which suggest great difficulties for an unambiguous treatment, even probabilistic, of these dynamics, and thus for capturing them by any scientific model, especially mathematical–experimental one. The latter, even those of QLTs, are unlikely to predict the effects of these dynamics, unless their richness and ambiguities are reduced to simplified opinions, as those in the Clinton–Gore experiment. Such opinions emerge from this richness, but this emergence is disregarded, allowing QLSs to work and be effective vis-à-vis classical models. It is possible that some of this complexity could be handled by non-mathematical scientific theories, which is, however, far from certain either. Literature, on the other hand, offers an alternative, partly philosophical, way of handling and enacting, as Eliot’s poem does (also in representing his decision in writing it), this complexity and helping us to confront it and estimate, *qualitatively*, its effects, while allowing for *discursive ambiguity*, precluded in human decision sciences, including QLSs, by the unambiguity principle [18,19].

As a reflection of this difference, I should like to consider an iconic text of modernist literature, Jorge Louis Borges’s ‘Pierre Menard, the author of the *Quixote*’. In Borges’s story, Cervantes’s *Don Quixote* becomes a new *Pierre Menard’s* text, while being word-by-word identical to the original:

Those who have insinuated that Menard devoted his life to writing a contemporary *Quixote* besmirch his illustrious memory. Pierre Menard did not want to compose *another* Quixote*,* which surely was easy enough—he wanted to compose *the* Quixote. Nor, surely, need one be obliged to note that his goal was never a mechanical transcription of the original; he had no intension of *copying* it. His admirable ambition was to produce a number of pages which coincided—word for word and line for line—with those of Miguel de Cervantes. [45, p. 91]

The story becomes an allegory of a complex, including in its ambiguity, functioning of language, keeping in mind that language also has a capacity for being unambiguous. As stressed earlier, no unambiguity, even mathematical one, could have emerged otherwise. In illustrating, the essential *difference* between Menard’s and Cervantes’s ‘identical’ texts, Borges’s *text* says:

It is a revelation to compare Menard’s Don Quixote with Cervantes'. The latter, for example, wrote (part one, chapter nine):

. . . truth, whose mother is history, rival of time, depository of deeds, witness of the past, exemplar and adviser to the present, and the future’s counselor.

Written in the seventeenth century, written by the ‘lay genius’ Cervantes, this enumeration is a mere rhetorical praise of history. Menard, on the other hand, writes:

. . . truth, whose mother is history, rival of time, depository of deeds, witness of the past, exemplar and adviser to the present, and the future’s counselor.

History, the mother of truth: the idea is astounding. Menard, a contemporary of William James, does not define history as an inquiry into reality but as its origin. Historical truth, for him, is not what has happened; it is what we judge to have happened. [45, p. 43]

This is not a matter of a correct reading of Cervantes’s novel, to the degree one could speak of *a* correct, reading it, let alone *the* correct one, which is impossible. This is an illustration of the uniqueness of each literary composition, defined in its meaning by a writing or a reading, while allowing for and even implying an ambiguity of its meaning, in a broad sense of the impossibility of containing and controlling this meaning. Menard arrived at his *decision* to reproduce *the text of Don Quixote*, as against *Cervantes’s novel*, which cannot be copied as *Cervantes’s novel*. The latter will always remain unique to Cervantes. Every reading of a text or any construction of the author’s experience in this reading makes it a new, unique event of experience, governed by the free will principle. The novel, thus reauthored, becomes unique each time, akin to (if not as creative as) the original creation of a work of literature or art, defined by an immense space of decisions offered by thinking and language, which literature and art exemplify, perhaps maximally.^20^

This situation is a manifestation of the capacity for ambiguity inherent in language and it is retained in the compositional abstractions or refinements of literature, as against, mathematics and science, which require the unambiguity principle. Insofar, as it represents a new mathematical concept or theory, a mathematical text does not, in general, repeat an earlier text, although it can repeat some elements of an earlier concepts and recombine them in a new composition, without assuming its Platonist origin in some pre-existent primordial domain. By the unambiguity principle, however, a mathematical or scientific text must have a more fully shared mathematical or scientific meaning, which disregards a different additional meaning it can have for an individual person. There can be no Pierre Menard, the author of the *Elements* of Euclid, *as a mathematical text*, or of the *Principia* of Sir Isaac Newton, *as a physical text* (which is also a mathematical text). There are personal components in one’s experience of these texts or were in their creations, but they are bracketed in their mathematical or scientific functioning*,* although they may enter other types of readings, such as historical or biographical, or our experiences of their mathematics or sciences. Conversely, there may be shared elements in our experience of literature and art, but they coexist, on equal footing, with the unique aspects of our experience, defined by the free will principle.

The unambiguity principle is not required for the functioning of literature and art and tends to inhibit their functioning. The free will principle is by contrast crucial. A new work of art is a bet on its future, never certain at the time of its creation, because future responses to this work by others are governed by the free will principle. This is also true about mathematical or scientific concepts or theories, such as Galois’s concept of group or Galois theory, Riemann’s concept of manifold, Heisenberg’s concept of matrix variables in QM or his QM itself, or Bohr’s complementarity. In these cases, however, the unambiguity principle strictly applies, as it must in mathematics and science, or other theoretical disciplines, such as philosophy, but not in literature and art. They thrive on ambiguity, ultimately in the sense, assumed in this article, of the impossibility of assigning definitive properties to a literary or artistic work. This impossibility allows for the possibility, by the free will principle, of changing the meaning of literary texts or objects of other arts in each experience, such as that of Cervantes himself versus Menard in Borges’s story, or real readers of Cervantes. In addition, writing is also always a reading of what one writes, as in my writing this sentence. Of course, I aim at making this sentence unambiguous, as is required by the scientific and philosophical nature of my article, as opposed to a novel or a poem.

The question is within what limits a science can describe or predict the complexity of human thinking and decision making that literature and art represent and, along with other human endeavours, such as philosophy, or mathematics and science, enact. To address, at least in in terms of reasonable predictions, this complexity is the ultimate task of human decision sciences. If, I argue, their mathematics could do so, it must be extraordinarily complex as well, possibly more complex than what we need for reaching the next stage in fundamental physics [43]. It may be beyond anything that we can imagine now, although it may already exist in mathematics itself. It is, again, also conceivable that science will no longer be a mathematical–experimental project, as Feynman thought might happen one day. Perhaps mathematics, too, will no longer be mathematics as we understand it now. Part of my argument here is that QT changed the use of mathematics, albeit without changing the nature of mathematics itself, but instead affirming its abstract nature. Perhaps, both mathematics and science will be more like literature or art. This is a tantalizing possibility. Even as a possibility, it may require us to rethink the nature of mathematics and science, or conversely, literature and art, which may in turn be more mathematical and scientific than we think. It is the future and new ways of thinking that it may bring that is most at stake and that casts its shadow over present-day mathematics and science, or literature and art, but only a shadow, which keeps this future uncertain and difficult to predict. This shadow makes the future of mathematics and science, or literature and art, *quantum-like*. But then, this is equally true about human life: it invites us to live in the uncertain shadow of the future rather than in the overdetermined shadow of the past as we tend to do, declining or missing this invitation, which is also an invitation to freedom in all life endeavours.

## Data Availability

This article has no additional data.

## Footnotes

^1^
Although the uses of ‘realist’ and ‘ontological’ sometimes diverge, they are close and will be used interchangeably here. Another term for realist theories is ‘ontic’, derived, as ontological, from the ancient Greek *ontos* (being).

^2^
Quantum phenomena are assumed here to be defined by the fact that in considering them the Planck constant, *h,* must be taken into account. I put aside qualifications of this definition, which are not germane to this article (e.g. [3, pp. 37–38]). See also [4, pp. 52–56].

^3^
Bohr changed his views, sometimes significantly, arriving at his ultimate, strong RWR, interpretation around 1937. This requires one to specify to which version of his interpretation one refers. I shall do so as necessary, while focussing on his ultimate interpretation, unavoidably in the present *interpretation* of his interpretation. The designation ‘the Copenhagen interpretation’ requires even more qualifications as concerns whose interpretation it is, say, that of W. Heisenberg, P. Dirac, or J. von Neumann, which compels me to avoid this designation.

^4^
See [1] for a discussion of the mathematical topology of the formalism of both QM and QLTs. The article does not consider complementarity and the decision structure defining it, although both complementarity and the role of decision may be seen as physically grounding the topology of the formalism of QM, as I argue here.

^5^
Such conceptions of physical or mental reality have been advanced since Plato, the most famous proponent of this view, or even the pre-Socratics, such as Parmenides, who inspired Plato, and they have persisted since and still do.

^6^
There are arguments denying even a relative freedom of our decisions, and thus any free will, such as, those for ‘superdeterminism’. These arguments, which are marginal and controversial, will be put aside here.

^7^
The present view does, however, assume the existence of a single physical universe and hence is different from the many worlds interpretations of QM. In question in the present view are local transformations of physical reality by quantum experiments, the set-up of which defines these transformations. One can be concerned with the quantum aspects of the universe, for example, at its pre-big-bang stage, but only on the basis of a locally obtained evidence. One cannot experiment with the universe as a whole by interfering into its overall state from the outside.

^8^
The argument offered in the section builds on earlier works by this author [18,19] but revises them by considering the unambiguity and the free will principles, not discussed there.

^9^
An observer, as an agent, is sometimes considered as a quantum system, usually under the assumption that all physical systems considered are quantum, as discussed in [24,25].

^10^
There are cases, such as ’the Wigner’s friend‘ experiment, involving human subjects as objects of investigation by QM. These cases would require adjusting this claim, without, however, invalidating it, because the experiments considered are essentially defined by the role of physical quantum objects in them [25].

^11^
There are psychoanalytic and philosophical arguments, outside the quantum-like framework, in which the unconscious is considered along RWR lines. See [27] and references therein.

^12^
I put aside the question of these capacities in some animals, which is not germane in considering QT and QLTs.

^13^
This is a new principle in QLTs, although there are works, including by this author, in accord with it [18,19,30,31]. The works, however, relate human thinking and QM conceptually rather than mathematically.

^14^
I put aside some technical mathematical aspects of quantum-like treatment of this experiment, considered in, among other works [33], which has further references. These nuances would not affect my argument.

^15^
If one instantly repeats the same measurement the outcome is commonly assumed to be the same. This assumption is, however, an idealization which does not affect the uniqueness of each quantum event [3, pp. 213–218, 335].

^16^
There are concepts of complementarity in classical physics, but they are different from that of Bohr [30, p. 19].

^17^
In this respect, the Clinton–Gore experiment is similar to Bohmian mechanics, where complementarity variables, such as those of position and momentum, could be defined simultaneously, but never measured simultaneously (giving the same predictions as those of QM). Bohmian-like theories are used in human decision science, where their physically problematic features, such as incompatibility with relativity, play no role (e.g. [34, pp.184–192].

^18^
For an instructive analysis see [33].

^19^
One can use a technological device as an ‘agent’ initiating, or registering outcomes of, experiments, or even generating numerical predictions. Any such device has, however, to be ultimately set up by a human agent.

^20^
Addressing literature or language along QLT lines is uncommon, although there have been some commendable, if thus far, limited attempts, e.g. [46–49]. How effective QLTs are in dealing with literature, in all its cognitive richness and ambiguity, is the question, the same question I am asking about human experience, which literature captures and helps us to understand, possibly exceeding the capacities of scientific or even philosophical theories in this regard.
